# Performance of Polymer Electrolyte Membrane for Direct Methanol Fuel Cell Application: Perspective on Morphological Structure

**DOI:** 10.3390/membranes10030034

**Published:** 2020-02-25

**Authors:** Hazlina Junoh, Juhana Jaafar, Nik Abdul Hadi Md Nordin, Ahmad Fauzi Ismail, Mohd Hafiz Dzarfan Othman, Mukhlis A. Rahman, Farhana Aziz, Norhaniza Yusof

**Affiliations:** 1 School of Chemical and Energy Engineering, Faculty of Engineering, Advanced Membrane Technology Research Centre (AMTEC), Universiti Teknologi Malaysia, UTM Skudai 81310, Johor Bahru, Malaysia; hazlina.junoh@gmail.com (H.J.); afauzi@utm.my (A.F.I.); hafiz@petroleum.utm.my (M.H.D.O.); mukhlis@petroleum.utm.my (M.A.R.); farhana@petroleum.utm.my (F.A.); norhaniza@petroleum.utm.my (N.Y.); 2 Department of Chemical Engineering, Universiti Teknologi PETRONAS, Seri Iskandar 32610, Perak, Malaysia; nahadi.sapiaa@utp.edu.my

**Keywords:** polymer electrolyte membranes (PEMs), proton and methanol transportation, membrane morphology, direct methanol fuel cells, porous electrolytes, fuel cells

## Abstract

Membrane morphology plays a great role in determining the performance of polymer electrolyte membranes (PEMs), especially for direct methanol fuel cell (DMFC) applications. Membrane morphology can be divided into two types, which are dense and porous structures. Membrane fabrication methods have different configurations, including dense, thin and thick, layered, sandwiched and pore-filling membranes. All these types of membranes possess the same densely packed structural morphology, which limits the transportation of protons, even at a low methanol crossover. This paper summarizes our work on the development of PEMs with various structures and architecture that can affect the membrane’s performance, in terms of microstructures and morphologies, for potential applications in DMFCs. An understanding of the transport behavior of protons and methanol within the pores’ limits could give some perspective in the delivery of new porous electrolyte membranes for DMFC applications.

## 1. Introduction

The development of renewable energy to combat the environmental impact of fossil fuels has gained great attention in recent years. Solar energy, wind energy, geothermal energy, bioenergy, hydropower, ocean energy and fuel cells are among the most well-known renewable energy sources that could potentially replace dependency on fossil fuels. Among these, fuel cells are one of the favorable sources due to their modular design, small footprint and portability [1]. Their advantages have been demonstrated by the emerging demand for high capacity portable batteries and electric vehicles that utilize compact fuel cells [1]. The development of fuel cells, such as the phosphoric acid fuel cell (PAFC), molten carbonate fuel cell (MCFC), solid oxide fuel cell (SOFC), alkaline fuel cell (AFC), proton exchange membrane fuel cell (PEMFC) and direct methanol fuel cell (DMFC) can be distinguished by their chemical reactions, operation process conditions and types of electrolytes, respectively [1]. Among these, the DMFC is more practical due to its ability to run at low operating temperatures and a high theoretical energy density compared to PAFCs, MCFCs and SOFCs, which operate at temperatures of 150–220 °C, 650 °C, and 1000 °C, respectively [2,3,4,5]. Moreover, fuel storage and transportation happen at ambient conditions, making DMFC much more effective when compared to AFCs and PEMFCs, which both require high-pressure hydrogen gas storage.

**Figure 1:**
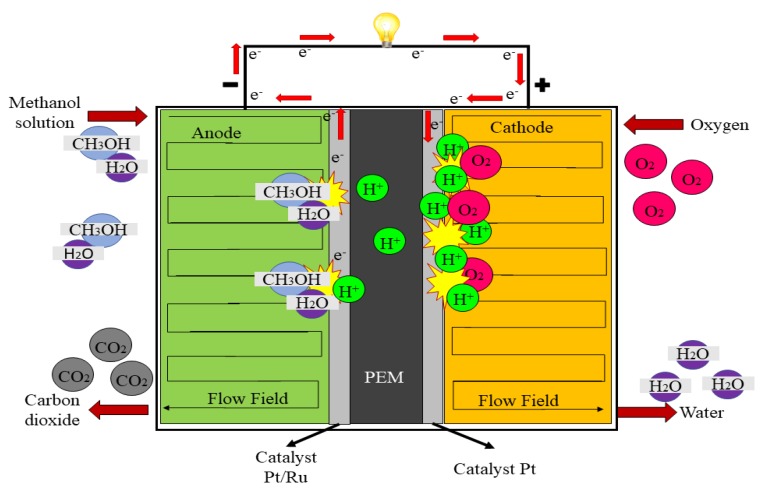
Basic structure of the direct methanol fuel cell (DMFC) system.

Principally, the performance and efficiency of the DMFC system are both greatly affected by the operating temperature, pressure and methanol concentration, and one should know the limitations that exist. The important aspects that should be taken into consideration are the slow oxidation rate, electrolyte membrane stability under operating conditions (60–90 °C), as well as excellent gas and water management at the anode and cathode sides, respectively [6]. Membrane stability refers to the chemical and mechanical strength of the electrolyte membrane itself, which under the DMFC’s operating conditions is not expected to undergo collapse or defect. The core preparation for electrolyte membranes in the DMFC application should consist of free proton (H^+^) ions. This may refer to the acid electrolyte or acid polymer that can provide a pathway for protons to be transferred from the anode to the cathode side. Thus, these types of electrolyte membranes are called “proton exchange membranes (PEMs)” which serve as a way to transfer H^+^ from the anode to the cathode across the membrane effectively [2]. Figure 1 illustrates the basic structure of the DMFC system.

As shown in Figure 1**,** the methanol solution is fed on the DMFC anode side. The hydrogen atom will become electrochemically oxidized into protons, electrons and carbon dioxide upon reaching the catalyst. The protons will diffuse through the PEM and will eventually react with the oxide ion (from the oxygen atoms) at the cathode side to form water molecules, while the electrons will travel to the external circuit to complete the energy demand [2]. The side products, such as CO_2_ and H_2_O, give a plausible advantage of DMFCs since the CO_2_ can be converted into useful products, such as methanol, formic acid and hydrocarbon [7,8], while the H_2_O can be reused for other applications. The reactions at the anode and cathode side in the DMFC system are as presented below.

Anode: CH_3_OH + H_2_O → CO_2_ + 6H^+^ + e^−^

Cathode: ^3^/_2_O_2_ + 6H^+^ + 6e^−^ → 3H_2_O

Overall reaction: CH_3_OH + ^3^/_2_O_2_ → CO_2_ + 2H_2_O

As matter of concern, a promising PEM should possess the inherent properties that will enable it to operate efficiently in the DMFC system [9,10,11,12]. These inherent properties consist of: (1) high proton conductivity (≥0.05 Scm^−1^), (2) acceptable mechanical strength and stability, (3) high chemical and electrochemical stability under operating conditions (60–90 °C), (4) extremely low fuel crossover (<5 × 10^−6^ moles min^−1^ cm^−2^), (5) low water transport through diffusion and (6) acceptable electro-osmosis and the capability for fabrication into the membrane electrode assembly (MEA) [13].

However, the main concerns that have been critically studied in recent years are low proton conductivity (at 60–90 °C) and methanol crossover, which have restricted the Nafion^®^ capability in the DMFC system. With that being said, methanol crossover was the main issue controlled by PEM. This is due to the composition of the membranes in nature that enables both proton diffusion and methanol permeation [14]. The same transport mechanisms possessed by methanol molecules and proton ions makes it more likely that they depend upon each other [15]. Since methanol is fed on the side of the anode, the concentration gradient induces a transfer of mass from the anode to the cathode, which eventually leads to an over-potential electrode on the side of the cathode. On the cathode side, the methanol that crosses the membrane will react directly with the oxygen. As the methanol brings the electrons directly while crossing the membrane, the power produced by the fuel cell system eventually decreases.

The methanol crossover has indeed made a huge impact on DMFC performances since it can reduce the capability of DMFC in providing high power generation. This phenomenon was realized by Heinzel and Barragán [16] who, in their study, found that there are five major parameters influencing methanol crossover which are: (1) methanol concentration, (2) operating temperature, (3) pressure (4) membrane thickness and (5) catalyst morphology. Nevertheless, since the solid polymer membrane (Nafion) was applied in their work, they have neglected the factor of the membrane materials and structure, which can be one of the significant parameters that need to be taken into consideration. For instance, the less hydrophobic and hydrophilic regions within the membrane matrix could provide a higher tortuosity for methanol transport, which shows excellent fuel barrier properties, as well as a massive dispersion of ion clusters for proton transport across the membrane. Since then, the electrolyte membrane fabrication has significantly improved in order to resolve the aforementioned problems regarding proton conductivity, methanol barrier properties and whit standability of the fabricated PEMs under operation conditions (60–90 °C). The evolutions of PEMs started from the modification of perfluorinated ionomer membranes, followed by the introduction of fluoropolymer-based membranes, non-fluorinated polymer membranes and acid–base blend membranes. 

The modification of perfluorinated ionomer membranes is done by introducing materials such as silicon, palladium, montmorillonite and alumina. Fluoropolymer membranes, such as poly tetrafluoroethylene, poly (ethylene-alt-tetrafluoroethylene), poly (vinylidene fluoride), perfluorosulfonic acid (PFSA), poly(ethylene-alt-tetrafluoroethylene) (ETFE), poly (vinyl fluoride) (PVF) and poly (fluorenyl ether) (PFE) also have been evaluated for their performance in DMFC applications. Newly non-fluorinated polymer membranes, such as sulfonated polyimides, polystyrene sulfonic acid, sulfonated poly (ether ether ketone), poly (vinyl alcohol), sulfonated poly (arylene ether sulfone), polyphosphazene, polybenzimidazole and sulfonated polysulfones have gained a great deal of attention in past decades due to the ease of their modification to meet the required performance. In addition, the acid–base blended polymer has been introduced to stimulate a good separation of proton and methanol in DMFC applications. Interestingly, the introduction of composite electrolyte membranes appears to have a positive impact on their performance in DMFC. The composite electrolyte membranes consist of the main polymer and inorganic fillers, such as silica, heteropolyacid, zirconium phosphate (ZrP), montmorillonite, zirconium hydrogen phosphate, metal oxide, single-walled carbon nanotubes and so on, which increase the performance of DMFC when compared to the pristine membranes [17,18,19,20,21,22,23,24,25,26]. 

However, the development of electrolyte membrane fabrication was mainly designed to meet the requirements of the excellent performance of DMFC with high proton conductivity (>0.05 Scm^−1^) and low methanol permeability (<10^−6^ moles min^−1^ cm^−2^) under the DMFC operating conditions [27]. Having said that, these membranes possess considerably low proton conductivity values due to their restricted, densely packed structure which limits the water uptake and free water transportation through the membrane matrix [28]. Therefore, the development of new porous-type electrolyte membranes with nanoscale pores is believed to improve the membrane efficiency and physicochemical properties of the cell, as opposed to the dense electrolyte membrane. Jiang et al. [29] did pioneering work on the porous structure of sulfonated poly (ether ether ketone) (SPEEK); they found that the porous structure does help in collecting more water and inducing proton transportation as a result of the easier pathways created by the pores. This was proven when they found that the value for proton conductivity of porous SPEEK (58 mScm^−1^) was slightly higher than dense SPEEK (~49 mScm^−1^) membranes. With regards to water uptake, the calculation of proton conductivity is a key parameter which is greatly affected by the structural morphologies of the membranes [30]. Higher porosity, narrower pores and a more compact distribution of the ionic cluster in a polymer matrix are, therefore, excellent qualities for proton conductivity [31], while methanol crossover could be suppressed [32]. However, when dealing with an open, porous structure, the probability of methanol crossover due to the kinetic separation that occurs when the pore size is slightly higher than the kinetic diameter of the methanol molecules cannot be neglected. This paper, therefore, aims to discuss the common membrane structures that were practically developed in a lab-scale to examine their effects on the performance of DMFC, as well as to explore the possibilities and challenges of porous structure that would offer excellent performance for DMFC applications.

## 2. Morphology of PEMs

The type of fabricated membranes for DMFC applications can be defined by their structural aspect—either dense, thin and thick, layered, sandwiched, pore-filling or porous membranes. In other words, a membrane is defined as a solid matrix that consists of distinct pore sizes. These pore sizes have specific nomenclatures, as recommended by International Union of Pure and Applied Chemists (IUPAC), which are macroporous (pore diameters > 50 nm), mesoporous (2 nm < pore diameters < 50 nm), microporous (0.2 nm < pore diameters < 2 nm) and nonporous (pore diameters < 0.2 nm) [33]. Generally, membrane morphology can be divided into two groups: dense and porous. The standpoints for these membranes’ morphologies are discussed explicitly in this paper.

### 2.1. Dense Electrolyte Membranes

Dense membranes are commonly defined as homogeneous membranes with no pores at the limits of electron microscopy. According to IUPAC, these membranes possess pore diameters of less than 0.2nm [16,33]. The dense membrane transport mechanism mainly covers the driving force of the pressure concentration and the electrical gradient potential [34,35,36]. Nonetheless, in electrolyte membrane processing, the electrical potential gradient mostly acts as the driving force for transportation of either cations or anions from the feed stream to permeate the sides. In other words, the PEMs with fixed charged ionic carriers on the polymer backbone act as semipermeable barriers which only allow the oppositely charged ions to pass through [37]. 

**Figure 2:**
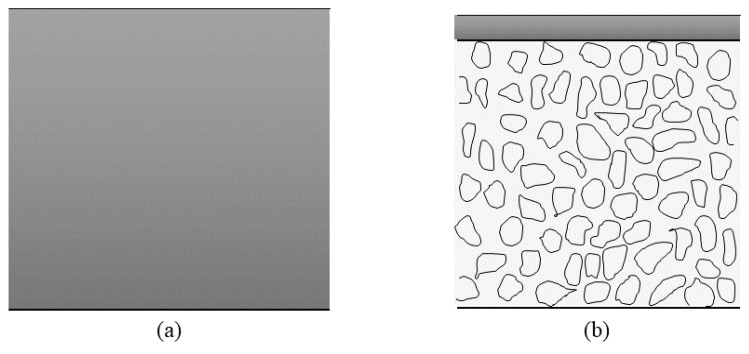
(**a**) Symmetric and (**b**) asymmetric membrane structure.

**Figure 3:**
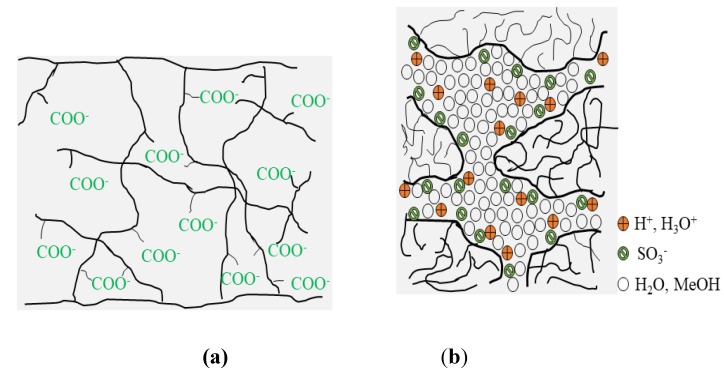
(**a**) Typical electrically charged membrane and (**b**) electrically charged Nafion membrane.

Essentially, PEMs have an asymmetric structure rather than a symmetric structure (Figure 2). According to Baker [38], the first breakthrough in the asymmetric membrane was done by Loeb and Sourirajan in 1962, in order to improve the performance of the dense symmetric membrane. Subsequently, in past decades, much more research and development (R&D) has been done in fabricating asymmetric membranes for DMFC applications. These asymmetric membranes consist of a dense or thin top skin layer for selectivity and thicker support layers of a microporous structure. Typically, for fuel cell applications, an electrically charged membrane was characterized by a very finely microporous structure when the pores were carrying fixed positively or negatively charged ions [38]. Figure 3b illustrates the structure of the most common electrical electrolyte membranes that are governed by DMFC applications. 

As mentioned above, the nature of the transport of proton and methanol is concurrent, as water molecules act as a means of transport. The distinction between proton and methanol transport depends on the molecular size that can penetrate through the membrane’s pores when all other factors are neglected in the study. Even though the Nafion possesses a higher proton conductivity, the methanol barrier properties are lower due to the huge distinction between the hydrophobic and hydrophilic regions. Novel non-fluorinated membranes with lower hydrophobic and hydrophilic regions have been identified to address the problem of methanol permeation. For instance, Othman et al. [39] studied the effect of membrane morphologies toward methanol and proton transportation behavior within a sulfonated poly (ether ether ketone) (SPEEK) membrane. In their study, they found that the SPEEK membranes offer a much lower separation of the hydrophobic and hydrophilic domains due to the smaller hydrophobic backbone, which allows more branch formation inside the SPEEK matrix. Moreover, these branches provide a higher tortuosity for methanol transport within the SPEEK matrix which eventually reduces the methanol crossover. Despite this, the conductivity of the proton decreased to an acceptable range, compared to Nafion, due to the highly packed sponge-like structure. 

Later, Ilbeygi et al. [40] studied the properties of organic–inorganic nanocomposite membranes for DMFC, which consisted of sulfonated poly (ether ether ketone) (SPEEK), Cloisite15A^®^ clays and triaminopyrimidine (TAP). In their study, they found that the methanol permeability for SPEEK/Cloisite15A^®^/TAP (0.52 × 10^−6^ cm^2^ s^−1^) is lower compared to Nafion 117 (4.29 × 10^−6^ cm^2^ s^−1^) at 60 °C, respectively. This phenomenon is attributed to the distinctions between the hydrophilic and hydrophobic regions of the polymer structure. More branches of the SPEEK polymer backbone limit the flexibility (lesser hydrophobic and hydrophilic regions) and, in addition to Cloisite15A^®^ clay, give merit to the methanol barrier properties with narrowed channels. A complete exfoliated nanocomposite SPEEK/Cloisite15A^®^/TAP membrane could provide a tortuosity pathway for methanol to permeate. Significantly, these membranes successfully managed to increase the methanol barrier properties, however, they reduced the proton conductivity (47 mScm^−1^) to an accepted limit range due to the highly packed sponge-like structure.

### 2.2. Thin and Thick Electrolyte Membranes

Thin and thick membranes mainly refer to the difference in membrane thickness, neglecting the micropores of the membranes. The structure of these membranes is identical to the dense membranes. Many studies have been reported on the effect of the membrane’s thickness on proton conductivity, especially for DMFC applications. Most recently, Aricó et al. [27] wrapped up some of the intrinsic properties that must be controlled for electrolyte membranes to be used in DMFC applications. One of the important properties that can alter the performance of DMFC is the membrane’s thickness. Membrane thickness could affect the proton conductivity since a thicker membrane offers more resistance for proton transport with lower methanol barrier properties [27].

Nevertheless, a thinner membrane produces a lower fuel utilization due to the higher methanol crossover, despite its higher performance in DMFC [41]. On the other hand, the solution viscosity plays an important role in membrane formation since it can largely affect the membrane’s thickness. For instance, Yee et al. [42] conducted an experimental study on SPEEK membranes with different concentrations. In their study, they varied the concentrations of SPEEK based on 10–30 wt.% of DMAc. Moderate thickness in the range of 30–50 µm was attained with the optimum concentration of 15–25 wt. %. Despite that, this moderate thickness offered proton conductivity in the range of 10^−3^ Scm^−1^ which can be considered low for DMFC applications. However, having said that, thickness in the range of 30 < μm < 100 is preferred for PEMs in DMFC applications when compared to commercial Nafion^®^ membranes (> 100 μm).

### 2.3. Layered Electrolyte Membranes

The layered electrolyte membrane refers to a membrane with another material residing on the top layer of the membrane’s surface. Simply put, this layered electrolyte membrane can be fabricated from the same or different materials which stick together. The layered structure aims to block the methanol crossover, while maintaining or increasing the proton conductivity. This happened regarding a well-known problem with single membranes—that they are prone to swell at high operating conditions, which allows methanol crossover that eventually retards the performance of DMFC. 

A higher methanol crossover current density (125–150 mAcm^−2^ at 60 °C and 250–300 mAcm^−2^ at 90 °C) on single-layered Nafion 117 encouraged Shao and Hsing [43] to discover new methods in delivering a lower methanol crossover without jeopardizing the proton conductivity value. In their study, they have successfully fabricated three different types of layered membranes consisting of Nafion 112 and poly (vinyl) alcohol (PVA) which are: (1) Nafion 112/Nafion 112/Nafion 112; (2) Nafion 112/Nafion 112-PVA/Nafion 112 and (3) Nafion 112/sulfonated Nafion 112-PVA/Nafion 112. Also, they found that a layer-by-layer membrane composition consisting three combinations of Nafion 112 resulted in a higher methanol crossover when compared to Nafion 112/Nafion 112-PVA/Nafion 112. The PVA helps in blocking methanol due to its smaller hydrophilic and hydrophobic regions, which are plausible with a layered membrane which provides a more tortuous pathway for methanol to pass through. Moreover, a Nafion-layered membrane consisting of sulfonated polyvinylidene fluoride-co-HFP (PvdF-co-HFP) and polybenzimidazole (PBI) was fabricated by Mondal et al. [44] via the dip-coating method. From their work, the Nafion 117/SPvdF-co-HFP/PBI membranes presented a higher power density (39 mW/cm^2^ at 0.2 V) than single-layered Nafion 117 (36 mW/cm^2^ at 0.2 V).

**Figure 4:**
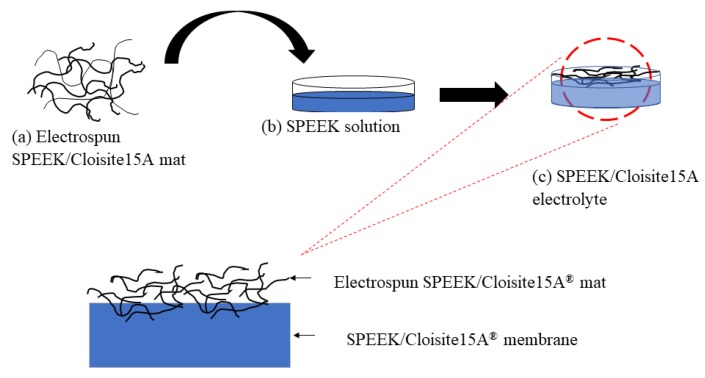
Fabrication step of layered electrospun sulfonated poly (ether ether ketone) (SPEEK) Cloisite15A® nanocomposite electrolyte membrane.

Another method has also been proposed for layered membrane fabrication, which is the combination of electrospinning and casting techniques for electrolyte membranes. The potential of nanofibers as ion exchange membranes for fuel cell applications has been elucidated by Zhang et al. [45]. This was proven through experimental analysis by Awang et al. [46], who produced electrospun SPEEK/Cloisite15A^®^ nanofiber for DMFC applications. In their study, they found that the value for proton conductivity and methanol permeability were 12 mScm^−1^ and 11.87 × 10^−8^ cm^2^s^−1^, respectively. Figure 4 illustrates the fabrication step for electrospun SPEEK/Cloisite15A^®^ electrolyte membranes. 

Other studies on layered membranes to improve DMFC performance have also been conducted by other researchers. [47,48,49,50]. However, the proton conductivity value was said to be considerably low (12 mScm^−1^) for these membranes when compared to the prerequisite value (>0.05 Scm^−1^). This phenomenon occurs as a result of the dense membrane structure which restricts the proton transportation across the membrane. The tight structure allows the blocking of methanol permeation; however, the proton also finds it quite difficult to pass through. The Grotthuss (proton hopping) mechanism is the only mechanism that has been applied for this proton transportation, rather than the vehicle (freely moving proton) mechanism, which limits the proton transportation. 

### 2.4. Sandwiched Electrolyte Membranes

**Figure 5:**
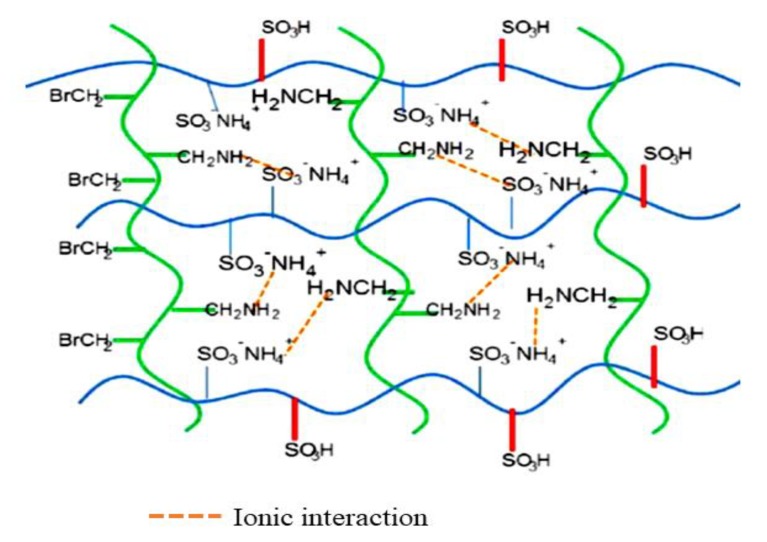
Backbone structure of the sulfonated poly (2,6-dimethyl-1,4-phenyleneoxide) /brominated poly (2,6-dimethyl-1, 4-phenyleneoxide) /sulfonated poly (2,6-dimethyl-1,4-phenyleneoxide) (SPPO/BPPO/SPPO) membranes and ionic interaction at the center interface. Copyright © 2014 Elsevier B.V. All rights reserved.

In contrast to layered electrolyte membranes, these sandwiched electrolyte membranes are fabricated when one membrane is squeezed in between two membranes made of different materials. The selection of materials to be squeezed is done in order to improve the performance of the parent membranes for DMFC applications. For instance, Bakangura et al. [51] studied sulfonated poly (2,6-dimethyl-1,4-phenyleneoxide) /brominated poly (2,6-dimethyl-1, 4-phenyleneoxide) /sulfonated poly (2,6-dimethyl-1,4-phenyleneoxide) (SPPO/BPPO/SPPO) membranes as sandwiched composite electrolyte membranes for DMFC applications. The proton conductivity was found to be slightly lower than the SPPO single membrane at 100% relative humidity (RH), which was 0.135 Scm^−1^ and 0.142 Scm^−1^, respectively. This phenomenon happened likely due to the structure of BPPO, which rendered the proton transportation at the center (Figure 5). However, the methanol permeability showed remarkable improvement when compared to SPPO single membrane and Nafion 117 due to the formation of the ionic crosslinking of the acid–base polymer.

Moreover, Li et al. [52] also incorporated sandwiched membranes in their study on DMFC performance. In their work, sulfonated holey graphene oxide (SHGO) paper was squeezed in between sulfonated poly (ether ether ketone) (SPEEK) membranes. The obtained proton conductivity was higher (155.6 mScm^−1^) than single SPEEK and Nafion 112 (138.6 mScm^−1^) membranes. Thus, it is proven that the high density of sulfonic groups allows for the higher diffusivity of protons in the membrane matrix. The methanol barrier of the SPEEK/SHGO membrane (7.05 × 10^−6^ cm^2^s^−1^) was slightly lower than Nafion 112 membranes (15.40 × 10^−6^ cm^2^s^−1^). The main contributor to these phenomena is the higher mechanical stability (less swelling behavior) of the prepared membranes when compared to Nafion 112 due to the strong interfacial and van der Waals interactions between SPEEK/SHGO and SHGO’s graphitic planes, respectively.

**Figure 6:**
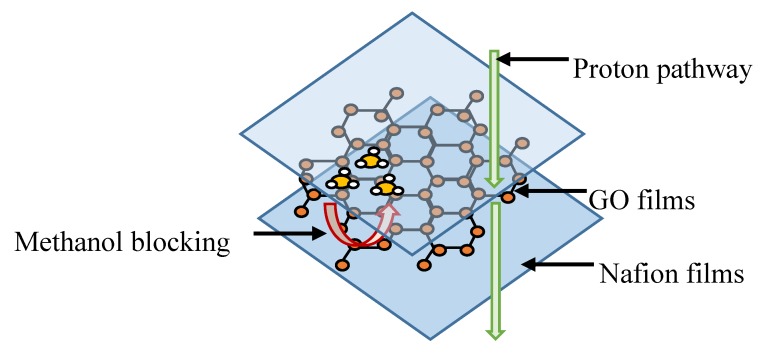
Sandwiched membrane consisting of Nafion–graphene oxide (GO)–Nafion.

Furthermore, in a study by Yan et al. [53], they found that the selectivity of graphene oxide (GO)’s crystalline structure repelled the methanol transportation, while allowing the transportation of protons. Figure 6 illustrates the graphene oxide (GO) film being sandwiched by Nafion membranes. The alteration of pristine Nafion membranes is mainly done to decrease the methanol permeability, while increasing or maintaining the proton conductivity value. Aside from the specialty possessed by the Nafion membrane in delivering high proton conductivity, the hydrogen bond developed between the oxygen group in GO and the water molecules could retain more ionic channels for proton transportation [54]. It has also been reported that the selectivity of a GO membrane is more dominant for water species than organic compounds, such as methanol. Thus, the repulsive behavior for methanol is greater than water, which could limit the transportation of methanol in GO membranes. However, the inclusion of GO by the sandwiching method also forfeited the proton conductivity by 7% at 80 °C when compared to pristine Nafion [53]. The decrease in proton conductivity is said to be caused by the formation of cracks during the membrane fabrication process and the difference in the size of the sandwich materials. However, due to their improved performance compared to the pristine membrane structure, these sandwiched membranes attracted a lot of attention from other researchers, including Dewi et al. [55].

### 2.5. Pore-Filling Electrolyte Membranes

**Figure 7:**
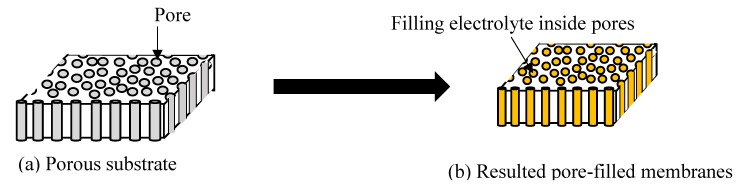
Pore-filled membranes. (**a**) Porous substrate; (**b**) Resulted pore-filled membranes

In essence, the pores of a porous substrate (Figure 7a) will be filled with an electrically charged electrolyte to construct a pore-filling electrolyte membrane, as illustrated in Figure 7b. The host membrane or substrate should have a higher mechanical strength in order to prevent swelling during the impregnation process. Having said that, the formation of porous membranes by phase inversion techniques mainly depend on both aspects of the thermodynamic and kinetic factors of the polymer, solvent, and non-solvent [56]. Next, for selectivity purposes, the porosity of the membrane is considered to be an important factor in the delivery of a good matrix for composite pore-filled membranes [56].

The key to the morphology of the membrane is the type of solvent/non-solvent. Different solvents/non-solvents give a different type of morphology. N-methylpyrrolidone (NMP), N, N-dimethylacetamide (DMAc), N-N-dimethylformamide (DMF) and dimethysulfoxide (DMSO) are commonly used as the solvents for membrane fabrication. These solvents will, essentially, induce finger-like structures in the membrane when immediately immersed in a coagulant bath (non-solvent). On the other hand, a solvent with a low affinity to the non-solvent, such as tetrahydrofuran (THF) could provide a spongy structure with a dense or partially porous top layer [56]. To prove this, Nguyen and Wang [56], in their study, synthesized porous co-polyimide from different monomers of 3,3′,4,4′-biphenyltetracarboxylicbdianhydride (SBPDA), p-phenylenediamine (PPDA) and 4,4′-oxydianiline (ODA) using NMP as a solvent. However, in their study, to get the sponge-like structure, 1-butanol was used as a non-solvent in order to delay the demixing process. This demixing process refers to the solvent and non-solvent exchange rate which will affect the membrane morphology. Delayed demixing will provide the membrane with lower porosity, fewer finger-like structures and a smaller mean pore size [57].

Other than that, from their study, they found that the time of immersion in the coagulant bath (non-solvent) also affects the membrane morphology. The increased time in the coagulant bath resulted in a larger mean pore size. The same phenomenon happened when a different solvent evaporation time was applied for wet/dry phase inversion techniques regarding the delayed demixing process. Salim et al. [58], in their study on poly ether sulfone (PES)/oxygenated graphitic carbon nitride (OGCN)-hydrophilic surface modifying macromolecules (LSMM), found that the delayed demixing process, by relaxing the casting solution before immersion takes place, resulted in larger pore sizes as the solvent evaporation time increased.

The first breakthrough in the area of pore-filling electrolyte membranes was made by Yamaguchi et al. [59] in DMFC applications. In their study, they observed that the commercial porous polyimide (PI) incorporated with sulfonated poly ether sulfone (SPES) possessed a much lower methanol permeability when compared to Nafion 117. This phenomenon occurred because of the nature of the substrate, which is capable of limiting the free transport of water and providing a low swelling behavior due to the possession of the hydrophobic regions. Even though the selectivity of these pore-filling membranes is higher compared to Nafion 117, the obtained proton conductivity was found to be a moderate value (20^−2^–30^−2^ Scm^−1^). Consequently, these pore-filling electrolyte membranes have caught the attention of other researchers, especially Alwin et al. [60] and Khabibulin et al. [61], due to its specialty towards methanol barrier properties and proton conductivity.

Subsequently, the incorporation of inorganic nanofiller inside the substrate’s pore in order to increase the proton conductivity of pore-filled electrolyte membranes was given attention by Pandey et al. [62]. In their work, they used zirconium phosphate (ZrP) as an inorganic filler for poly (vinylidene fluoride) (PvdF) substrate. The incorporation of ZrP inside PVDF’s pores was done via in situ growth techniques. A two-step fabrication was conducted as follows: (1) the porous PvdF was immersed into zirconium ion (Zr^4+^) precursor solutions for 5 h at 80 °C and (2) the impregnated PvdF/Zr^4+^ membrane was placed into H_3_PO_4_/HCI solutions for 48 h. Simultaneously, the pore-filled PvdF/ZrP membranes were then placed inside the oven for at least 12 h at 120 °C. In their work, they found that the proton conductivity, which was 5.2 mScm^−1^ when compared to room temperature (1.25 mScm^−1^) is dominant at higher temperatures (60 °C), but the methanol permeability at 30 °C (4.1 × 10^−7^ cm^2^s^−1^) was lower when compared to Nafion 115 (11.8 × 10^−7^ cm^2^s^−1^) and Nafion 117 (12.8 × 10^−7^ cm^2^s^−1^), which means the performance of the PvdF/ZrP composite membrane is greater than the commercial Nafion membrane.

Based on the categorized electrolyte membranes’ types, it must be noted that all these membranes can be differentiated based on their configurations, but possess the same dense, sponge-like morphological structure. Even though the methanol permeability of these membranes is enhanced when compared to the pristine Nafion, the proton conductivity is still lower, as previously recorded. Thus, in order to enhance the proton conductivity as well as the methanol barrier properties, one should understand the transport of protons and methanol within the pores.

## 3. Characterization of PEMs

Essentially, there are four main parts to determining the effectiveness of PEMs in DMFC and these are: (a) conductivity properties; (2) permeation of DMFC species; (3) thermal and mechanical stability; (4) morphology and elemental analysis and (5) DMFC single test performance. These characterizations will help researchers in determining and optimizing PEMs as promising candidates in the DMFC system. The perspectives of these characterizations are discussed explicitly in this paper.

### 3.1. Conductivity Properties

The conductivity properties are mainly based on the ion exchange capacity (IEC), water uptake and proton conductivity of the PEMs. Previously, Yang et al. [63] conducted an experiment on the effect of IEC on the proton conductivity of pristine sulfonated poly sulfone (SPSU), composite SPSU- graphene oxide (GO), as well as a hybrid SPSU-functional polymer brush and modified graphene oxide (FPGO). In their study, they discovered that both SPSU–GO and SPSU–FPGO possessed lower IEC values, which were 1.23 and 1.21 mmol/g, respectively. However, the pristine SSPU membrane showed a higher IEC value of around 15% to 17% when compared to both SPSU–GO and SPSU–FPGO. In conclusion, two factors that affected the IEC value in this study were, namely: (i) the introduction of FPGO, which diluted the concentration of sulfonic acid groups in the cross-linked membranes and (ii) the compact matrix of SPSU–FPGO, which limited the number of protons being transferred, especially at room temperature [63].

Nonetheless, the introduction of inorganic filler within the polymer matrix will affect the water uptake of the prepared membranes when compared to the pristine membrane. The addition of these inorganic fillers will increase the number of hydrophilic sites, such as OH^−^, COO^−^ and O^−^. For instance, both SPSU–GO and SPSU–FPGO possessed a higher water uptake when compared to pristine SPSU. However, the water uptake of SPSU–GO (50.7%) was higher than SPSU–FPGO (42.9%) membranes due to the strong interfacial interaction of covalent cross-linking, which makes SPSU–FPGO possess a compact network that makes it difficult for water to pass through [63].

In addition, Tawalbeh et al. [64] studied the effect of graphene oxide (GO) on pristine Nafion 117. In their study, they discovered that the proton conductivity of Nafion 117/GO (0.052 S/cm) composite membrane was lower when compared to pristine Nafion 117 (0.066 S/cm). These values of proton conductivity are believed to result from the agglomeration of layered GO within parent Nafion 117, which can block the proton conduction. Thus, proper techniques or methods to hinder the main problem regarding inorganic materials, which tend to attach with each other due to strong interfacial bonding, need to be thoroughly studied to achieve higher proton conduction. Furthermore, Barique et al. [65] conducted an experimental study on the effect of temperature and relative humidity (RH) on the proton conductivity of PEMs. In their study, they found that at 80 °C operating temperature, 60% and 80% RH resulted in conductivities of 0.05 and 0.1 S/cm for Nafion NR212 membranes, respectively. In addition, at low RH, they found that the proton conductivity did not show any remarkable increment until it reached 60% RH. It is noteworthy that the proton conductivity of Nafion NR212 remains identical with respect to the operating temperature between 80 and 100 °C [65].

### 3.2. Permeations of DMFC Species

Moreover, the permeability of methanol is one of the key components of the DMFC system and can determine the effectiveness of the system itself. A lower methanol value (< 10^−6^ moles min^−1^ cm^−2^) [66] is vital in order to meet the demand of an excellent performance of DMFC. For instance, the introduction of a hydrophobic cluster of PVdF–HFP and Cloisite 30 clay layers provided a larger tortuosity pathway for methanol to passing through the matrix of SPEEK/PVdF–HFP/Cloisite 30. As the value of PVdF–HFP loading increased from 5 to 20 wt.%, the methanol permeability decreased from 8.61 × 10^−7^ cm^2^ s^−1^ to 1.35 × 10^−7^ cm^2^ s^−1^ [67]. Explicitly, the methanol uptake of the PEMs should be low, which indicates the lower affinity of PEMs toward methanol. However, the sorption of water and methanol through ionic channels are simultaneously affecting the barrier properties [68]. Thus, the chosen materials for PEMs are important to hinder methanol crossover, yet deliver a good or high proton conductivity.

### 3.3. Mechanical and Thermal Stability

Swelling behavior is one of the characteristics that needs to be hindered as it can affect the methanol barrier properties of PEMs particularly at the standard operating temperature range (60–120 °C) of the DMFC system. For example, the introduction of a compact cross-linked network of SPSU/FPGO-1 by the inorganic filler FPGO has prevented structural changes in polymer hybrid membranes when compared to neat SPSU. These composite PEMs showed remarkable values of dimensional stability at a temperature of 70 °C [63]. In addition, the mechanical stability of PEMs can be considered as one of the important parameters which need to be studied in DMFC applications. For instances, the loading of inorganic fillers within the parent polymer matrix may have an impact on the mechanical strength of the PEMs. The higher loading of inorganic fillers may be of a highly fragile nature due to the compact structure of inorganic fillers (most likely due to agglomeration). In a study by Sonpingkam and Pattavarakorn [69], they found that a small amount of inorganic filler (lower than 3 wt.%) enhanced the tensile strength of pristine sulfonated poly (ether ether ketone) (SPEEK) membranes when compared to loading an amount higher than 3 wt.%. Thus, a completely homogeneous polymer and inorganic nanofiller mixture is important for the delivery of a good separation of inorganic filler within the membrane matrix, which eventually increase the mechanical stability of the membrane.

**Figure 8:**
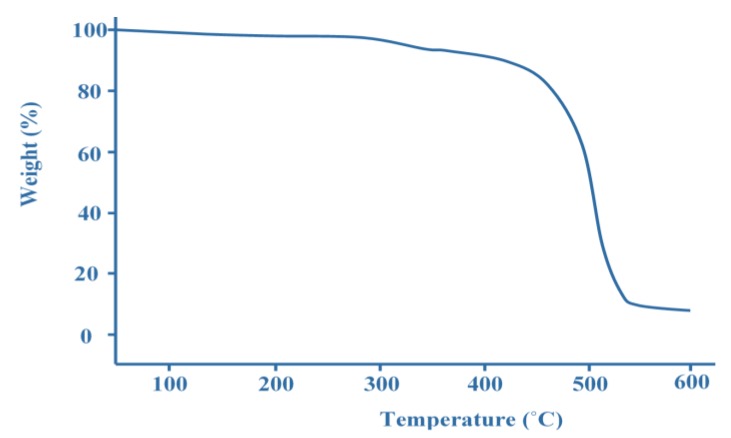
Thermogravimetric analyzer (TGA) curve for Nafion 112.

In order to be an excellent candidate, the glass temperature (Tg) and the first degradation of materials in PEMs should exceed the operating temperature of the DMFC, which is between 60 °C and 120 °C. In general, the Tg of materials in PEMs can be defined by differential scanning calorimetry (DCS), while the step degradation of materials can be measured by the thermogravimetric analyzer (TGA). Normally, the scanning rate for TGA testing is about 10 °C/min for a better transition resolution. Typically, all membranes experienced three stages of degradation [5]. For example, Figure 8 illustrates the TGA curve for commercial Nafion 112.

As shown in Figure 8, the first stage of degradation happens in the temperature range of 50–300 °C followed by the second stage and third stage degradations at the region of 300–400 °C and 400–500 °C, respectively. During the first degradation, the uptake is mostly related to moisture capture inside the Nafion 112 matrix. Later, the second stage indicates the degradation of the sulfonic acid group and, finally, the third stage is associated with the degradation of the main chain of Nafion 112. It can also be deduced, from Figure 8, that Nafion 112 can withstand a DMFC system operating at temperatures between 60 to 120 °C, as there was no significant weight loss of up to 300 °C.

### 3.4. Morphology and Elemental Analysis

The homogeneity of materials in the PEM matrix is important, as it can affect the selectivity of PEMs, especially for the transport of proton and methanol, in particular for composite PEMs. Good compatibility and homogenous distribution of materials in the PEM matrix are, therefore, very crucial for excellent PEMs in the DMFC system. Essentially, there are a few standard characterizations that can be implemented in order to ensure the compatibility and homogenous distribution, namely: (1) surface morphology and elemental analysis; (2) phase separation analysis and (3) structural changes in PEMs. These characterizations will be explained thoroughly in this subtopic.

Recently, Pagidi et al. [70] studied the surface morphology and elemental analysis of a PTFE–ZrP–PVA composite membrane. The study found that the continuity and uniform distribution of ZrP-PVA observed at the top layer of the PTFE membrane were strongly related to the resulting proton conductivity and methanol permeability values of 0.0281 Scm^−1^ and 14.5 × 10^−7^ cm^2^s^−1^, respectively. These values showed remarkable improvement when compared to Nafion 117. To further confirm the presence of ZrP on the PTFE surface, an energy dispersive X-ray spectrometer (EDX) analysis was used. More than half of the elemental weighted percent (%) of the surface was covered by zirconium and phosphate, values of which were 31.9% and 26.44%, respectively.

**Figure 9:**
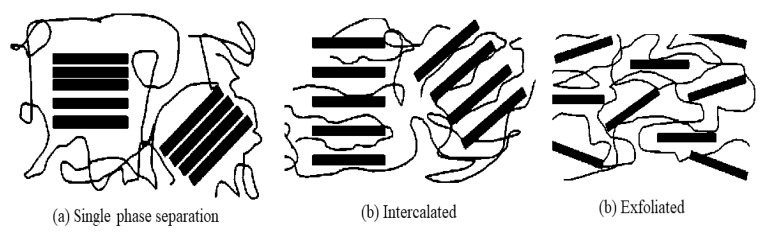
Phase separation in composite polymer electrolyte membranes (PEMs), such as (**a**) single phase separation, (**b**) intercalated separation and (**c**) exfoliated separation.

The phase separation of composite PEMs can be mostly defined by single-phase separation, intercalated and exfoliated. The single-phase separation occurs when the polymer chains are unable to act as a divider for the inorganic layers, which eventually depletes the proton conductivity and methanol barrier properties. The intercalated separation occurs when the polymer chain manages to act as a divider between inorganic layers and eventually produces a multi-layered interchange of polymer and inorganic layers. Lastly, the exfoliation occurs when the polymer chains are separated by individual inorganic layers with an average distance from each other. Figure 9 illustrates all the separation types between inorganic filler and parent polymer chain.

**Figure 10:**
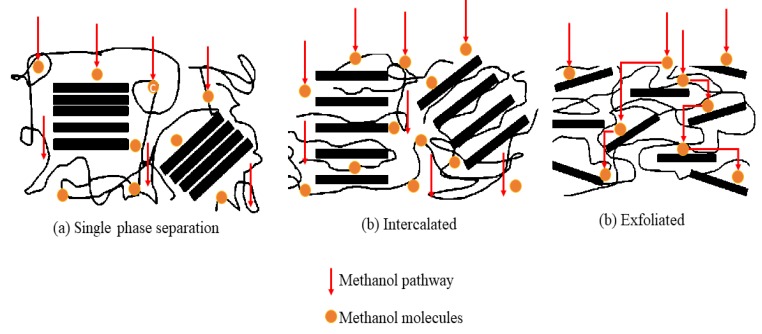
Mechanism of methanol transportation through different separations of composite PEMs.

For instance, Divya et al. [50] studied the effect of molybdenum disulfide (MoS) in Chitosan membranes for DMFC applications. In their study, they found that MoS provided an exfoliated separation in the chain of Chitosan, which eventually led to higher proton conductivity and methanol barrier properties of 2.92 mS/cm and 3.28 × 10^−8^ cm^2^/s, respectively. The separation categories can be seen from the X-ray diffraction (XRD) analysis. From the XRD result, the strong peak indicated the strong intercalation of the inorganic filler in the polymer matrix, which elucidates that the PEMs possessed an exfoliated phase separation in the inorganic layers and polymer chains. This type of separation is important in delivering a good performance in PEMs for DMFC applications. Therefore, as the exfoliation separation formed, the mechanism for transporting methanol would be tortuous rather than straight forward, as shown in Figure 10. From Figure 10, the mechanism transportation of methanol within single and intercalated separation in PEMs matrix are mostly identical when compared to exfoliated separation.

Furthermore, the surface changes of PEMs can be defined by surface roughness analysis via atomic force microscopy (AFM), which can affect the proton conductivity of PEMs. For instance, Neelakandan et al. [48] studied the effect of charge surface-modifying macromolecules (cSMM) toward the proton conductivity of pristine sulfonated poly (phenylene ether ether sulfone) (SPEES) membranes. In their study, they found that the nodular size of SPEES–cSMM was higher when compared to pristine SPEES, which increased the proton uptake up to 16.1 × 10^−3^ S/cm from 6.7 × 10^−3^ S/cm.

### 3.5. DMFC Single Cell

The single cell test was governed to conclude the DMFC performance test. In the DMFC single test, the cell voltage versus current density will be evaluated using a fuel cell analyzer system. Many studies have been carried out to evaluate the parameters that affect the DMFC performance, such as: (1) the applied pressure of the cathode and anode inlet; (2) the flow rate of the feed at anode and cathode; (3) methanol concentration; (4) applied voltage and (5) operating temperature. For instance, Ercelik et al. [71] studied the DMFC single test for the Nafion-based composite membrane. In their study, they found that the power density increased up to 612.96 W/m^2^ and gradually decreased as the current density increased (~2250 A/m^2^) for the Nafion membrane when 1 M methanol was used. This behavior is believed to be caused by the negative effect of methanol crossover and carbon monoxide (CO) poisoning at the anode side [71]. Table 1 summarizes some studies on the characteristics of PEMs for DMFC applications.

## 4. Transportation of Proton and Methanol within the Pores

**Figure 11:**
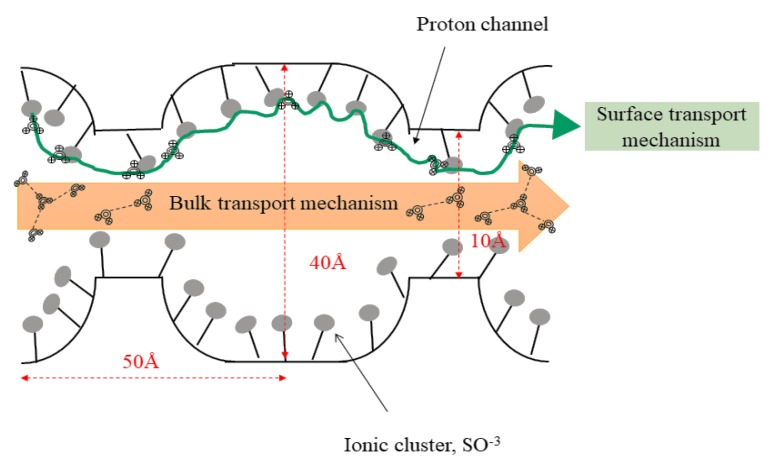
Transports mechanism inside a cluster model of Nafion.

As mentioned earlier, the electrically charged membranes are governed by very finely microporous structures when pores carry fixed positively or negatively charged ions. Figure 11 illustrates the transport mechanisms that occurs inside a cluster model of Nafion [81]. Essentially, the Grotthuss mechanism offers the diffusivity of protons through a fixed negatively charged ion on the polymer backbone, i.e., Nafion. On the other hand, the vehicle mechanism depends mainly on the free-water molecules as a transportation agent [82,83,84]. From Figure 11, it can be clearly observed that the transport process of a proton occurs simultaneously by bulk and surface transport mechanisms [40]. These bulk and surface transport mechanisms differ mainly by the Grotthuss and vehicle diffusivity mechanisms, respectively [40,81]. 

Surface transportation mainly occurs via the Grotthuss mechanism, where the protons diffuse by hopping from one ionic cluster (fixed negatively charged ion) to another without jeopardizing the chemical structure of the carriers (SO_3_^−^ and COO^−^ groups). On the other hand, the bulk transport mechanism is dominated by the vehicle mechanism since free-water molecules inside the membrane matrix act as transport agents. This type of bulk transportation will simultaneously change the chemical structure of the carriers. Nonetheless, at moderate operating conditions (< 120 °C and 1 atm), these free-water molecules also offer drawbacks in the transportation of methanol throughout the material which will eventually deteriorate the DMFC performance [85]. This is mainly due to the size and the inclusion of a 10 Å ionic channel which allows methanol with a lower kinetic diameter (3.8 Å) to pass through [86,87]. The selectivity toward methanol molecules through the membrane matrix is not preferred for DMFC to avoid poisoning at the cathode side [88]. 

In addition, Balsara and Beers [89] concluded that the ideal domain size for proton conductivity via vehicle mechanism is < 6 nm in width. As the pore size increased, the length domains of hydrophilic and hydrophobic regions also increased, which resulted in lower proton conductivity. Furthermore, Huang et al. [90] found that the proton conductivity increases as the porosity increases. However, this conclusion is still in its early stages and requires thorough experimental studies for a deeper understanding. Thus, a newly designed membrane with a completely porous substrate and narrower pore channels is crucial for addressing the problem of proton conductivity and methanol crossover.

## 5. Future Prospects in Electrolyte Membrane Fabrication

A new generation of porous membrane fabrication can be applied to the porous metal organic framework (MOF)-type membrane. The zeolitic imidazolate framework-8 (ZIF-8) seemingly has the potential to deliver high proton conductivity and methanol barrier properties, which have been described by Barbosa et al. [91] in their paper. Therefore, decreasing the average size of ZIF-8 crystals will increase the effective area, which can enhance the water uptake and eventually increase the proton conductivity [92]. This phenomenon is expected to be based on a rule of thumb where proton transportation mainly depends on the water molecules. In addition, due to the super hydrophobic behavior of ZIF-8, it is possible to retain water molecules inside the cage (the aperture size effect) by the hydrogen bonding of terminating imidazole linkers with water molecules, which eventually assist the proton transportation [92]. While the water molecules can also assist the transportation of methanol within the membrane matrix, these ZIF-8 membranes could offer a smaller aperture size (~3.4 Å) for methanol molecules (~3.8 Å) to enter, which simultaneously repels the methanol crossover [93].

**Figure 12:**
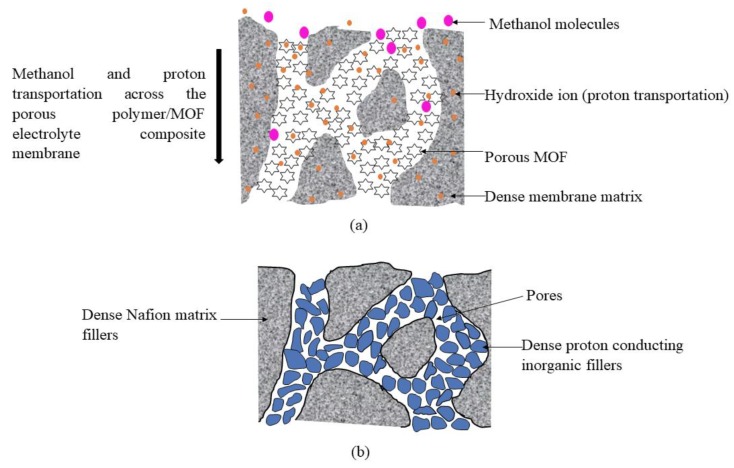
(**a**) Schematic diagram of proton and methanol conduction across porous polymer/MOF and (**b**) pore-filling type composite membrane of Nafion/inorganic fillers.

However, the use of a purely porous structure in direct methanol fuel cell applications is still in its early stages. With regard to the benefits of the porous electrolyte membrane structure and porous materials such as MOFs, it is widely expected that the combination of the two properties will enhance the conductivity of the proton and the tortuosity of methanol within the membrane matrix. The schematic diagram (Figure 12a) shows the possible interaction between proton conduction and methanol permeation across the porous polymer/MOF which is quite similar to the structure of the pore-filling Nafion/inorganic electrolyte composite membrane (Figure 12b) [94]. 

As illustrated in Figure 12, the difference between these two membranes, namely (a) and (b), is the size of the pores (being either mesoporous, microporous or microporous). If investigated in detail, the components within the pores of Nafion can only provide a microporous structure, contributed to by the space between the dense inorganic fillers/neighboring and inorganic fillers/Nafion matrix. The formation of these structures within the pores of Nafion leads to the dense structure of the membrane (Figure 12b) which can also limit the transportation of protons (only on the surface, rather than inside the inorganic filler). In contrast, Figure 12a shows the pores of the porous membrane filled with porous MOF, which means that the total configuration of the composite membrane in Figure 12a is a porous structure. The membrane selectivity is highly dependent on the membranes’ morphologies and porosities [95]. Thus, it can be concluded that a reduction in the opening size of MOF could hinder the permeation of methanol while, at the same time, allowing the transportation of protons within the pores of MOF and the membrane itself.

## 6. Conclusions

This paper focused on providing an insight into the structural effect of the electrolyte membrane matrix on proton conductivity and methane permeability. Most of the membranes tested offer a similar densely packed morphology, even with different configurations (dense, thin and thick, layered, sandwiched or pore-filling). The potential of pores in the delivery of high proton conductivity is dominated by water molecule transportation, yet the methanol could be suppressed by the size inclusion of the pores. Thus, it can be deduced that newly porous electrolyte membranes with smaller pore sizes could enhance the methanol barrier properties, while delivering high proton transportation in DMFC applications.

## Figures and Tables

**Table 1:** Polymer electrolyte membranes (PEMs) for direct methanol fuel cell (DMFC) applications.

PEMs	Proton Conductivity (mS/cm)	Methanol Permeability (× 10^−7^ cm^2^/s)	Water Uptake	Methanol Uptake	Swelling Ratio	Ref.
Poly (vinyl alcohol) /montmorillonite (PVA/MMT)	36.8	36.7	NA	NA	improved	[72]
Sulfonated poly (styrene-*b*-ethylene/butylenes-*b*-styrene) copolymer/Cloisite^®^Na^+^ (S-SEBS/Na^+^)	142	6.2	Relatively high	NA	NA	[73]
Sulfonated poly (arylene ether nitrile)/sulfonated graphene oxide (SPEN/SGO)	109	1.7	42.6	NA	13.57	[74]
Sulfonated poly (ether ether ketone)/Cloisite15A^®^/triaminopyrimidine (SPEEK/Cloisite15A^®^/TAP)	16.3	1.3	26.19	NA	NA	[75]
Sulfonated poly (ether sulfone)/graphene oxide (SPES/GO)	4.3	0.492	40.1	NA	NA	[76]
Nafion/Nanoporous carbon (Nafion/NPC)	75.1	9.8	NA	NA	NA	[77]
Sulfonated poly (cinlidene fluoride)/sulfonated magnetite @silica (sPVdF/sFe_3_O_4_@SiO_2_	64	20	33.6	NA	NA	[78]
Zeolitic imidazolate framework-carbon nanotube hybrid/sulfonated poly (ether ether ketone) (ZCN/SPEEK)	206	0.0245	40.2	NA	8.6	[79]
Zeolitic imidazolate framework-8/deoxyribonucleic acid (ZIF-8@DNA)	170	0.125	NA	NA	No swelling	[80]

NA: not available, not tested or studied.
